# Hybridized bands and stacking-dependent band edges in ferromagnetic Fe_3_GeTe_2_/CrGeTe_3_ moiré heterobilayer

**DOI:** 10.1038/s41598-022-08785-x

**Published:** 2022-03-24

**Authors:** Eunjung Ko

**Affiliations:** Korea Institute for Advanced Study, Seoul, 02455 Korea

**Keywords:** Physics, Condensed-matter physics, Electronic properties and materials

## Abstract

Owing to unique fundamental physics and device applications, twisted moiré physics in two-dimensional (2D) van der Waals (vdW) layered magnetic materials has recently received particular attention. We investigate magnetic vdW Fe_3_GeTe_2_ (FGT)/CrGeTe_3_ (CGT) moiré heterobilayers with twist angles of 11° and 30° from first-principles. We show that the moiré heterobilayer is a ferromagnetic metal with an *n*-type CGT layer due to the dominant spin-majority electron transfer from the FGT layer to the CGT layer, regardless of various stacked structures. The spin-majority hybridized bands between Cr and Fe bands crossing the Fermi level are found regardless of stacking. The band alignment of the CGT layer depends on the effective potential difference at the interface. We show that an external electric field perpendicular to the in-plane direction modulates the interface dipole and band edges. Our study reveals a deeper understanding of the effects of stacking, spin alignment, spin transfer, and electrostatic gating on the 2D vdW magnetic metal/semiconductor heterostructure interface.

## Introduction

2D vdW layered metal–semiconductor nonmagnetic heterostructures have been studied for developing high-performance devices with the contact of metal electrodes and semiconductors^1–7^. Recently, heterostructures consisting of 2D ferromagnetic (FM) vdW layered materials have attracted considerable interest^8–10^. In particular, research on the 2D vdW heterobilayer composed of magnetic metals and magnetic semiconductors is still early, as it was only recently revealed that vdW monolayer (ML) or few-layer materials retain magnetism^11–13^. Furthermore, the recent discovery of novel properties of twisted vdW nonmagnetic homo and heterobilayers^14–16^ further prompts the investigation of twisted vdW magnetic homo and heterobilayers.

Recent experiments have demonstrated the long-range 2D FM order along the out-of-plane direction in the metallic ML FGT^17–20^ and the semiconducting CGT bilayer samples^21,22^. Thus, metallic Fe_3_GeTe_2_ (FGT) and semiconducting CrGeTe_3_ (CGT) materials are promising candidates for forming low-dimensional heterobilayers. Several experimental works have reported that bulk FGT is an itinerant FM metal with a high Curie temperature, *T*_c_, of approximately 230 K; *T*_c_ can be increased to room temperature by electrostatic gating and decreased by decreasing the layer thickness^17–20^. Theoretical studies have reported changes in the magnetic anisotropy energy (MAE), magnetic order from the bulk to ML, the anomalous Hall effect, and spin-dependent transport in tunnel junctions^10,23–26^. On the other hand, some experimental studies have revealed that bulk CGT is an intrinsic FM semiconductor with a *T*_c_ of 61 K^27^ and an experimental bandgap, *E*_g_, in the range 0.2–0.7 eV^28–31^. Several theoretical works have examined variations in *T*_c_, magnetic order, MAE, and *E*_g_ from the bulk to ML^28,32–39^. Although each FGT and CGT has been thoroughly investigated, the basic properties of the FGT/CGT heterobilayer have not been fully explored. Moreover, there has not been much research on the twisted moiré heterobilayer of vdW magnetic metal/semiconductor materials^11^. Hence, it is worth examining the promising FGT/CGT twisted moiré heterobilayer.

The Schottky barrier heights at the interface between a metal and a semiconductor are crucial properties for device applications. They classify the heterostructure interface into a Schottky contact (*n*- or *p*-type) or an Ohmic contact. In general, the additional interface states caused by extra bonds at the interface facilitate the formation of Schottky contact^40,41^, whereas they complicate that of Ohmic contact. Thus, the Schottky barrier heights at the metal–semiconductor interface with Schottky contact have been extensively studied. Experimental studies have reported converting a Schottky contact to an Ohmic contact by large gating voltages in 3D metal/2D vdW semiconductor heterostructures^42,43^. Meanwhile, the formation of extra bonds at the interface between 2D vdW materials can be prevented due to the vdW interlayer distance, and thus the heterostructure has an abrupt interface. Because of this advantage, the band edges of heterostructures combined with 2D vdW metals and semiconductors have been investigated with and without electrostatic gating for device applications. Several theoretical studies have shown the electrostatic gating effect on the band edges of nonmagnetic 2D vdW metal/semiconductor heterobilayers^1,5^. However, first-principles studies on the band edges of 2D vdW magnetic twisted moiré metal/semiconductor heterobilayers have rarely been performed^11^.

In this work, we conducted a first-principles study on the structural, electronic, and magnetic properties of the FGT/CGT moiré heterobilayer with twist angles of 11° and 30°. First, we examined the atomic distortion and unfolded band structures in a large moiré supercell with a twist angle of 11°, and then investigated the band edges depending on stacking in a small moiré supercell with a twist angle of 30° with and without electrostatic gating. Each small moiré heterobilayer having one of six possible stacked structures was an FM metal with an *n*-type CGT layer, induced by the effective spin-majority electron transfer from FGT to CGT. We also compared the band structures of the FM and AFM heterobilayers, having FM and antiferromagnetic (AFM) spin alignments between FGT and CGT. We found that only the FM heterobilayer had the hybridized bands between the Cr and Fe atoms. Further, we explained the stacking-dependent band edges of the CGT layer in terms of the effective potential difference. Finally, we addressed the external electric field effect on the band edges of the CGT layer in the FM heterobilayer.

## Methods

We conducted first-principles electronic structure computations using the noncollinear density functional theory (DFT) with the Hubbard repulsion, *U*, and exchange interaction, *J*, as well as the spin–orbit coupling (SOC). We used the norm-conserving pseudopotentials^44^ with the SIESTA code^45^. We adopted the generalized-gradient approximation, parameterized by the Perdew–Burke–Ernzerhof (PBE) formula. Further, we used the rotationally invariant U approach^46^ to describe the electron correlation for Cr 3*d* orbitals. The atomic structures were optimized using the spin-polarized optB88 vdW method until all atomic forces were less than 0.001 eV Å^−1^ except for a huge moiré supercell with a twist angle of 11°, where the force criteria were 0.036 eV Å^−1^. In addition, we generated a semicore pseudopotential for Cr using the (3*s*^2^, 3*p*^6^, 3*d*^5^, 4*s*^0^) valence configuration; the valence configurations for Fe, Ge, and Te pseudopotentials were (3*d*^7^, 4*s*^1^, 4*p*^0^), (3*d*^10^, 4*s*^2^, 4*p*^2^), and (4*d*^10^, 5*s*^2^, 5*p*^4^), respectively. We generated real-space grids with a cutoff energy of 400 Ry and expanded the electronic wave functions using pseudoatomic orbitals (PAOs) of the double-zeta polarization basis set. We utilized a $16\times 16\times 1$ *k*-grid for all structure relaxation and electronic structure calculations except for a large moiré supercell with a twist angle of 11°, for which we applied a $2\times 2\times 1$ *k*-grid. For ML CGT, we obtained *U* = 5.97 eV and *J* = 1.00 eV for Cr 3*d* orbitals with a cutoff radius of 1.58 Å by using a PAO-based constrained DFT (cDFT) method^45,47^. However, our previous study confirmed that the suitable theoretical bandgap close to the experimental one was obtained by the PBE + U + SOC method using a *U* in the range of 3–4 eV and *J* = 1 eV^48^. Therefore, we selected *U* = 3.5 eV and *J* = 1 eV for our calculations. To remove the interactions between FGT and CGT, we applied a vacuum space of 16 Å.

## Results and discussion

### Twisted FGT/CGT moiré heterobilayer

Bulk FGT has a layered hexagonal structure with space group *P*6_3_/*mmc* and experimental hexagonal cell parameters *a* = *b* = 4.030 and 3.991 Å and *c* = 16.343 and 16.336 Å from refs. 47 and 48, respectively. In ML FGT, each unit cell contains three Fe atoms occupying two nonequivalent Fe positions; Fe1 and Fe2 denote the relevant atoms. ML FGT consists of five sublayers: the first and fifth are occupied by Te atoms, the second and fourth by Fe1 atoms, and the third by Fe2 and Ge atoms. Our relaxed lattice constants for pristine ML FGT that are obtained using the spin-polarized optB88 vdW method are *a* = *b* = 4.091 Å. Bulk CGT has a rhombohedral symmetry with space group *R*$\overline{3 }$ and experimental hexagonal cell parameters *a* = *b* = 6.8196 Å and *c* = 20.3710 Å at 5 K^27^. ML CGT has two Cr atoms in a unit cell. ML CGT consists of five sublayers: the first and fifth are occupied by Te atoms, the second and fourth by Ge atoms, and the third by Cr atoms. ML CGT has a honeycomb lattice of edge-sharing CrTe_6_ octahedrons, and Ge dimers located at the center of the hexagon. Our relaxed lattice constants for pristine ML CGT that are obtained using the spin-polarized optB88 vdW method are *a* = *b* = 6.940 Å. The point group for ML FGT and ML CGT is *D*_3h_ and *C*_1_, respectively. Thus the heterobilayer built by ML FGT and ML CGT has the *C*_1_ symmetry.

**Figure 1:**
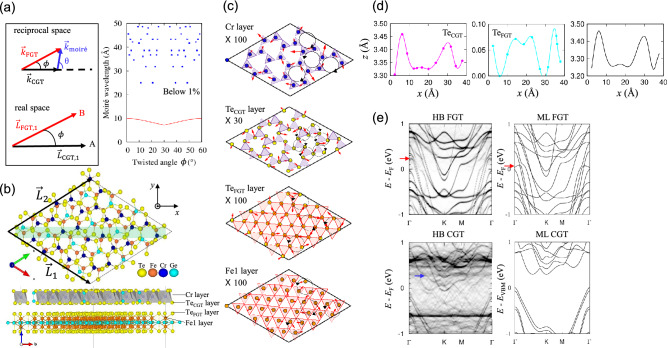
(**a**) $\overrightarrow{k}$ wave vectors of monolayer (ML) Fe_3_GeTe_2_ (FGT) and ML CrGeTe_3_ (CGT) in reciprocal space, moiré lattice vectors in real space (left panel), and moiré wavelength as a function of twist angles (red lines, right panel). (**b**) Top and side views of twisted moiré lattice with a moiré lattice constant of 25.02 Å, a lattice mismatch △ of 0.55%, a twist angle $\phi$ of 11.39°, and (*m*, *n*, *p*, *q*) = (1, 3, 3, 4). Only Fe1, Te_FGT_, Te_CGT_, Ge_CGT_, and Cr atoms near the interface are depicted in the top view. (**c**) In-plane displacement vectors (red arrows) of Cr, Te_CGT_, Te_FGT_, and Fe1 layers. The atomic structures are obtained using the spin-polarized optB88 vdW method. The red arrows are enlarged to 30 or 100 times their original size. The purple (red) triangles comprise the interface Te_CGT_ (Te_FGT_) atoms. (**d**) *Z*-coordinates of interface Te atoms along a diagonal in the *x*-axis (left and middle panels) and interlayer distance between Te_FGT_ and Te_CGT_ atoms (right panel). (**e**) Unfolded band structures of moiré heterobilayer calculated by the noncollinear PBE + U + SOC method when *U* = 3.5 eV and *J* = 1.0 eV. They are drawn along the unit-cell bandlines of FGT (HB_FGT) and CGT (HB_CGT). The moiré heterobilayer has a ferromagnetic (FM) spin alignment between the FGT and CGT layers. For comparison, the band structures of ML FGT and ML CGT are shown. The red and blue arrows indicate differences between the moiré heterobilayer and the monolayer.

The heterobilayer of ML FGT and ML CGT has a moiré pattern due to the lattice mismatch. In principle, the twist angle $\phi$ between the supercell lattice vectors of the FGT and CGT layers for a moiré supercell (i.e., $\phi$ between ${\overrightarrow{L}}_{\mathrm{CGT},1} \mathrm{and} {\overrightarrow{L}}_{\mathrm{FGT},1}$ in Fig. 1a) is the same as that between the unit vectors of ML FGT and ML CGT. Hence, the moiré reciprocal lattice vector, $\overrightarrow{k}$_moiré_, and the moiré wavelength, *λ*, can be easily obtained as $\vec{k}_{{{\text{moir}}\mathop {\text{e}}\limits^{\prime } }} = \vec{k}_{{{\text{FGT}}}} - \vec{k}_{{{\text{CGT}}}}$ and ${\uplambda } = \frac{{2{\uppi }}}{{\left| {\vec{k}_{{{\text{moir}}\mathop {\text{e}}\limits^{\prime } }} } \right|}}$, respectively, using reciprocal lattice vectors corresponding to the unit cell vectors. Concretely, when the unit vectors of ML CGT are ${\overrightarrow{a}}_{1}=a\left(\frac{\sqrt{3}}{2},-\frac{1}{2}\right)$ and ${\overrightarrow{a}}_{2}=a\left(\frac{\sqrt{3}}{2},\frac{1}{2}\right)$, and the unit vectors of ML FGT are rotated by $\phi$ relative to those of ML CGT, the reciprocal lattice vectors become ${\overrightarrow{k}}_{\mathrm{CGT}}=\frac{2\pi }{a}\left(\mathrm{1,0}\right)$ for ML CGT and ${\overrightarrow{k}}_{\mathrm{FGT}}=\frac{2\pi }{a(1-\delta )}\left(cos\phi ,sin\phi \right)$ for ML FGT, where $\delta =1-{a}_{\mathrm{FGT}}/{a}_{\mathrm{CGT}}$ as shown in the left panel in Fig. 1a. Thus, the moiré wavelength, *λ*, becomes ${\uplambda } = \frac{{2{\uppi }}}{{\left| {\vec{k}_{{{\text{moir}}\mathop {\text{e}}\limits^{\prime } }} } \right|}} = \left( {1 - \delta } \right)a/\sqrt {2\left( {1 - \delta } \right)\left( {1 - cos\phi } \right) + \delta^{2} }$^49,50^. The right panel in Fig. 1a shows *λ* (red lines) with respect to the twist angle $\phi$ between ML CGT and ML FGT optimized using the spin-polarized optB88 vdW method. The moiré superlattice vectors can be expressed as ${\overrightarrow{L}}_{\mathrm{CGT},1}=m{\overrightarrow{a}}_{1}+n{\overrightarrow{a}}_{2}$ and ${\overrightarrow{L}}_{\mathrm{CGT},2}=-n{\overrightarrow{a}}_{1}+(m+n){\overrightarrow{a}}_{2}$ for ML CGT and ${\overrightarrow{L}}_{\mathrm{FGT},1}=p{\overrightarrow{b}}_{1}+q{\overrightarrow{b}}_{2}$ and ${\overrightarrow{L}}_{\mathrm{FGT},2}=-q{\overrightarrow{b}}_{1}+(p+q){\overrightarrow{b}}_{2}$ for ML FGT, where *m*, *n*, *p*, and *q* are integers. We can obtain the commensurate moiré lattice vectors by applying a small biaxial strain △ between ML CGT and ML FGT layers. △ is estimated by $\Delta =|a\sqrt{{m}^{2}+{n}^{2}+mn}-b\sqrt{{p}^{2}+{q}^{2}+pq}|{(b\sqrt{{p}^{2}+{q}^{2}+pq})}^{-1}$. The moiré wavelengths within the constrained condition of △ being less than 1.0% are shown as blue squares in the right panel in Fig. 1a. Here the twist angle $\phi$ is determined as $cos\phi =\left({\left|{\overrightarrow{L}}_{CGT,1}\right|}^{2}+{\left|{\overrightarrow{L}}_{FGT,1}\right|}^{2}-{\left|\overline{AB}\right|}^{2}\right){\left(2\left|{\overrightarrow{L}}_{CGT,1}\right|\left|{\overrightarrow{L}}_{FGT,1}\right|\right)}^{-1}$, where $\left|\overline{AB}\right|=\left|{\overrightarrow{L}}_{CGT,1}-{\overrightarrow{L}}_{FGT,1}\right|=|m{\overrightarrow{a}}_{1}+n{\overrightarrow{a}}_{2}-p{\overrightarrow{b}}_{1}-q{\overrightarrow{b}}_{2}|$^51^.

Figure 1b displays the top and side views of the moiré superlattice with *λ* = 25.02 Å, △ = 0.55%, $\phi$ = 11.39°, (*m*, *n*, *p*, *q*) = (1, 3, 3, 4), and the total number of atoms being 352. Only Fe1, Te_FGT_, Te_CGT_, Ge_CGT_, and Cr atoms near the interface are depicted in the top view of Fig. 1b. The moiré supercell has CrTe_6_ hexagonal networks on top of FGT and various stacked structures. The in-plane and out-of-plane distortions obtained by the spin-polarized optB88 vdW method are displayed in Fig. 1c,d, respectively. The in-plane displacement vectors indicated by red arrows show more in-plane distortion in CGT than in FGT and are largest at the Te_CGT_ layer. In particular, the in-plane displacement vectors of all Te_CGT_ atoms have consistent patterns regardless of stacking, as depicted by black circles, but those of other atoms vary depending on stacking. The *z*-coordinates of Te_CGT_ and Te_FGT_ atoms inside the green shaded region in (b) vary depending on stacking, as shown in Fig. 1d. The out-of-plane distortion of the Te_CGT_ layer is much larger than that of the Te_FGT_ layer. The variation of interlayer distance between two interface Te layers is largest in the area where Te_CGT_ atoms are closely located above Te_FGT_ atoms.

The unfolded band structures of the moiré heterobilayer with a twist angle of 11.39° reveal some distinct features compared with the unit-cell band structures of a single layer, as shown in Fig. 1e. All the band structures were obtained using the noncollinear PBE + U + SOC method when *U* = 3.5 eV and *J* = 1.0 eV for Cr 3*d* orbitals, and an FM spin alignment between FGT and CGT was considered. The average magnetic moment is 3.64 ${\upmu }_{\mathrm{B}}$/atom for Cr, 2.61 ${\upmu }_{\mathrm{B}}$/atom for Fe1, and 1.65 ${\upmu }_{\mathrm{B}}$/atom for Fe2. The unfolded band structures of FGT (HB FGT) near the Fermi level are similar to the unit-cell band structures of ML FGT except for bands shifted higher in energy denoted by red arrows. On the other hand, the unfolded band structures of CGT (HB CGT) exhibit a reduced bandgap compared with that of ML CGT, and the conduction bands of HB CGT cross the Fermi level, generating an n-type CGT layer. Also, unexpected bands of HB CGT are observed as indicated by a blue arrow compared to ML CGT. These intriguing electronic features of moiré heterobilayer motivate us to examine the critical elements that influence band structures.

**Figure 2:**
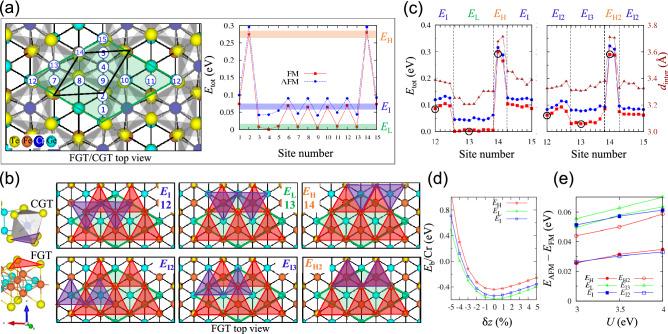
(**a**) Top view of FGT/CGT moiré supercell with a twist angle of 30° (green diamond) in the left panel, and total energy comparison of 15 stacked structures with the ferromagnetic (FM)/antiferromagnetic (AFM) spin alignment between the FGT and CGT layers in the right panel. The calculations are done using the spin-polarized optB88 vdW method. The supercell size is the same as the in-plane ML CGT unit cell size. A black diamond indicates the in-plane ML FGT unit cell. The numbered sites along two diagonals and one in-plane lattice indicate 15 stacked structures. The 15 stacked structures were made by sliding CGT over the numbered sites while FGT was fixed. The FM total energy is classified into three energies: *E*_I_, *E*_L_, and *E*_H_. (**b**) Six representative stacked structures of *E*_I_ (site 12), *E*_L_ (site 13), *E*_H_ (site 14), *E*_I2_, *E*_I3_, and *E*_H2_. The background lattice shows the FGT top view. The red (purple) triangles comprise the interface Te_FGT_ (Te_CGT_) atoms. Two purple triangles are situated above Fe1_FGT_ atoms for *E*_I_, Ge_FGT_ atoms for *E*_L_, and Te_FGT_ atoms for *E*_H_. *E*_I2_, *E*_I3_, and *E*_H2_ are obtained by rotating the CGT layer 180° and relaxing it. (**c**) FM/AFM total energy variation from *E*_I_ (*E*_I2_) through *E*_L_ (*E*_I3_) to *E*_H_ (*E*_H2_) representative structures with FM/AFM spin alignment and the interlayer distance, *d*_inter_, depending on FM stacked structures. The total energy and structures are obtained using the spin-polarized optB88 vdW method. The red, blue, and brown colors indicate FM, AFM, and *d*_inter_, respectively. (**d**) Binding energy, *E*_b_, per Cr atom of *E*_H_, *E*_L_, and *E*_I_ along the *z*-axis regarding small displacements. (**e**) Total energy difference between FM and AFM spin alignments obtained by using the noncollinear PBE + U + SOC method, when *U* is in the range 3–4 eV and the exchange interaction is *J* = 1.0 eV.

To investigate the electronic structures in terms of stacked structures, we constructed small moiré supercells of the FGT/CGT heterobilayer forming only one stacked structure with a twist angle of 30° and △ = 1.36%, as shown in Fig. 2a. The in-plane supercell indicated by a solid green diamond is equal to the $1\times 1$ in-plane unit cell of ML CGT and also corresponds to the 30° rotated $\sqrt{3}\times \sqrt{3}$ in-plane supercell of ML FGT, drawn by an open black diamond. Inside the supercell, there are two CrTe_6_ octahedrons.

ML FGT is an Ising-type FM metal, whereas ML CGT is an Ising-type FM semiconductor. To predict the magnetic structures of the heterobilayer, we compared the total energy of the heterobilayer with the FM and antiferromagnetic (AFM) spin alignments between ML FGT and ML CGT along the direction perpendicular to the interface. As shown in the left panel in Fig. 2a, we considered 15 different stacked structures while fixing ML FGT and sliding ML CGT. Concretely, we considered six different stacked structures in two diagonal directions of the in-plane supercell and three different stacked structures in the direction of the in-plane lattice. All the atoms were optimized during the relaxation in the *x-*, *y-*, and *z-*directions. The FM spin alignment between ML FGT and ML CGT always has lower energy than the AFM spin alignment at each stacked structure, as shown in the right panel in Fig. 2a.

We categorized the FM energy into three classes: the lowest, *E*_L_, intermediate, *E*_I_, and the highest energy, *E*_H_. The corresponding stacked structures are depicted in the top panel in Fig. 2b. The background lattice shows the FGT top view. The red and purple triangles represent the interface Te triangles in the FGT and CGT layers, respectively. We drew two purple triangles on the bottom Te atoms in CGT related to two CrTe_6_ octahedrons inside a supercell. In the *E*_I_ structure, the interface Te_CGT_ atom is above the Fe1_FGT_ atom; in the *E*_L_ structure, it is above the Ge_FGT_ atom; and, in the *E*_H_ structure, it is located above the interface Te_FGT_ atom. Three more possible stacked structures illustrated in the bottom panel were examined; the purple triangle consisting of interface Te_CGT_ atoms was rotated 180° relative to those in the top panel. Two more intermediate energy structures, *E*_I2_ and *E*_I3_, and another highest energy structure, *E*_H2_, were found. In *E*_I2_, *E*_I3_, and *E*_H2_ structures, the interface Te_CGT_ atom is above the Fe1_FGT_, Ge_FGT_, and Te_FGT_ atoms, respectively.

Figure 2c presents the total energy difference with the FM (red) and AFM (blue) spin alignments, as well as the interlayer distance (brown) between the interface Te atoms of the FM structures. All the atoms and axes were optimized during the relaxation in the *x-*, *y-*, and *z*-directions using the spin-polarized optB88 vdW method. The total energy difference changes gradually from *E*_I_ through *E*_L_ to *E*_H_ (left panel) and from *E*_I2_ through *E*_I3_ to *E*_H2_ (right panel). The total energy difference and the interlayer distance are the smallest for *E*_L_ and the largest for *E*_H_ and *E*_H2_. *E*_I_, *E*_L_, *E*_H_, *E*_I2_, *E*_I3_, and *E*_H2_ structures have interlayer distances of 3.39 Å, 3.22 Å, 3.78 Å, 3.33 Å, 3.31 Å, and 3.71 Å, respectively. At each FM heterobilayer, the lattice constants of the CGT and FGT layers become tensile strained and compressive strained, respectively, as summarized in Table 1. The total energy of FM spin alignment is lower than that of AFM for *E*_I_, *E*_L_, *E*_H_, *E*_I2_, *E*_I3_, and *E*_H2_ by 0.02 eV, 0.04 eV, 0.02 eV, 0.02 eV, 0.05 eV, and 0.03 eV, respectively. At the FM spin alignment, the total energy of the *E*_L_ structure is lower than that of *E*_I_, *E*_H_, *E*_I2_, *E*_I3_, and *E*_H2_ structures by 0.08 eV, 0.30 eV, 0.06 eV, 0.03 eV, and 0.29 eV, respectively. Figure 2d shows the binding energy, *E*_b_, per Cr atom along the *z-*direction regarding the small displacement. At the equilibrium position, the binding energy per Cr atom obtained using the spin-polarized optB88 vdW method has a negative value of − 0.44 eV, − 0.58 eV, and − 0.54 eV for *E*_H_, *E*_L_, and *E*_I_, respectively. The lower the total energy is, the lower the binding energy is. In the PBE + U + SOC method, the FM spin alignment also has lower energy than the AFM spin alignment when *U* is 3–4 eV and *J* = 1 eV for all the stacked structures, as shown in Fig. 2e.

**Table 1:** Lattice constant variation in the FM heterobilayer with a twist angle of 30°. During the relaxation of the heterobilayer, the lattice constants of CGT and FGT become tensile strained and compressive strained, respectively.

CGT	ML	*E*_I_	*E*_L_	*E*_H_	*E*_I2_	*E*_I3_	*E*_H2_
*a* (Å)	6.94	7.06	7.07	7.06	7.06	7.06	7.06
(%)		1.75	1.82	1.76	1.79	1.79	1.78

FGT	ML	*E*_I_	*E*_L_	*E*_H_	*E*_I2_	*E*_I3_	*E*_H2_
*a* (Å)	4.09	4.08	4.08	4.08	4.08	4.08	4.08
(%)		− 0.33	− 0.27	− 0.33	− 0.28	− 0.28	− 0.29

**Figure 3:**
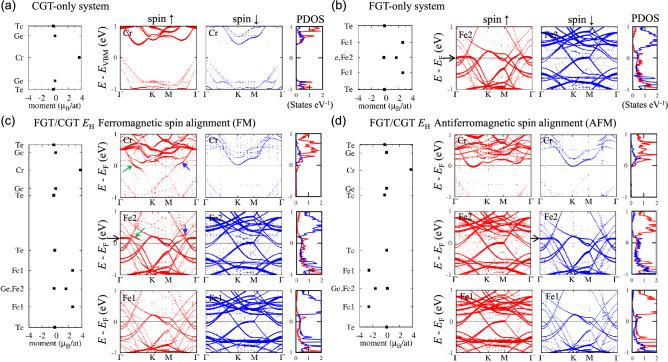
Magnetic moment (left panels), projected band structures (middle panels), and atom projected density of states (PDOS, right panels) of (**a**) the CGT-only system, (**b**) FGT-only system, (**c**) FM FGT/CGT heterobilayer of *E*_H_, and (**d**) AFM FGT/CGT heterobilayer of *E*_H_. The band structures are obtained by the noncollinear PBE + U + SOC method (*U* = 3.5 eV and *J* = 1.0 eV applied to Cr 3*d* orbitals). The band structures are projected onto a Cr or Fe atom; the color intensity corresponds to the projection magnitude. In (**c**), green and blue arrows indicate the hybridized bands between Cr and Fe2 atoms. In (**b**)–(**d**), the band change of Fe2 atoms near the $\Gamma$ point is indicated by black arrows.

The band alignment of FM and AFM heterobilayers was investigated. Figure 3 shows the magnetic moment (left panels), spin-majority (up) and spin-minority (down) band structures (middle panels), and atom projected density of states (PDOS, right panels) of (a) the FM CGT-only system, (b) FM FGT-only system, (c) FM FGT/CGT heterobilayer for *E*_H_, and (d) AFM FGT/CGT heterobilayer for *E*_H_ in the supercell band lines. The band structures were calculated by using the noncollinear PBE + U + SOC method on Cr 3*d* orbitals with *U* = 3.5 eV and *J* = 1.0 eV. It should be noted that the CGT (FGT)-only system refers to only the CGT (FGT) layer and no FGT (CGT) layer while maintaining the relaxed heterobilayer structures. The band projections onto the spin-up and spin-down bands of Cr or Fe atoms are distinguished by red and blue colors, respectively, with various color intensities proportional to the projection magnitude to examine the band alignment. The semiconducting bandgap of 0.43 eV is shown for a Cr atom in the FM CGT-only system (Fig. 3a), while the metallic band structure is shown for a Fe2 atom in the FM FGT-only system (Fig. 3b). The magnetic moment is 3.69 ${\upmu }_{\mathrm{B}}$/atom for Cr, 2.62 ${\upmu }_{\mathrm{B}}$/atom for Fe1, and 1.67 ${\upmu }_{\mathrm{B}}$/atom for Fe2. The atom PDOS of a Fe2 atom in (b) indicates an equivalent magnitude of spin-up and spin-down PDOS near the Fermi level. The CGT-only system features a conduction band minimum (CBM) at a k point between the $\Gamma$ and K points dominated by spin-up empty Cr 3*d e*_g_ bands. Its valence band maximum (VBM) at the $\Gamma$ point is dominated by Te 5*p* orbital characteristics, similar to the pristine ML CGT.

On the other hand, the heterobilayer formed by CGT- and FGT-only systems is an FM metal, as shown in Fig. 3c. The bands projected on a Cr atom show the initial Cr bands observed in the CGT-only system as well as unique spin-up hybridized bands with Fe2 bands crossing the Fermi level, as indicated by green and blue arrows, respectively. The hybridization is not related to Fe1 bands, as seen by the band projected on a Fe1 atom. Furthermore, when the hybridized spin-up bands are ignored, the CBM of Cr bands appears to be at the spin-down bands, located at an energy close to the Fermi level, generating an *n*-type CGT layer. The *n*-type CGT means that electrons are transferred from the FGT to CGT layers. In the right panel of Fig. 3c, the downward-band shifts of a Cr atom in the heterobilayer can be observed in the atom PDOS. The spin-up Fe2 bands shift higher in energy near the $\Gamma$ point when the system switches from an FGT-only system to a heterobilayer, as indicated by the black arrows in (b) and (c). These upward shifted spin-up Fe2 bands might also originate from the electron transfer from the FGT to CGT layers. Unlike the FM heterobilayer, the AFM-heterobilayer-band structures do not exhibit hybridized bands between FGT and CGT bands but do expose an *n*-type CGT layer via the spin-up and spin-down CBM of the original Cr bands crossing the Fermi level. AFM heterobilayer, like FM heterobilayer, features shifted spin-down Fe2 bands near the $\Gamma$ point that are higher in energy than those of the FGT-only system (black arrow in (d)), indicating electron transfer from the FGT to CGT layers.

**Figure 4:**
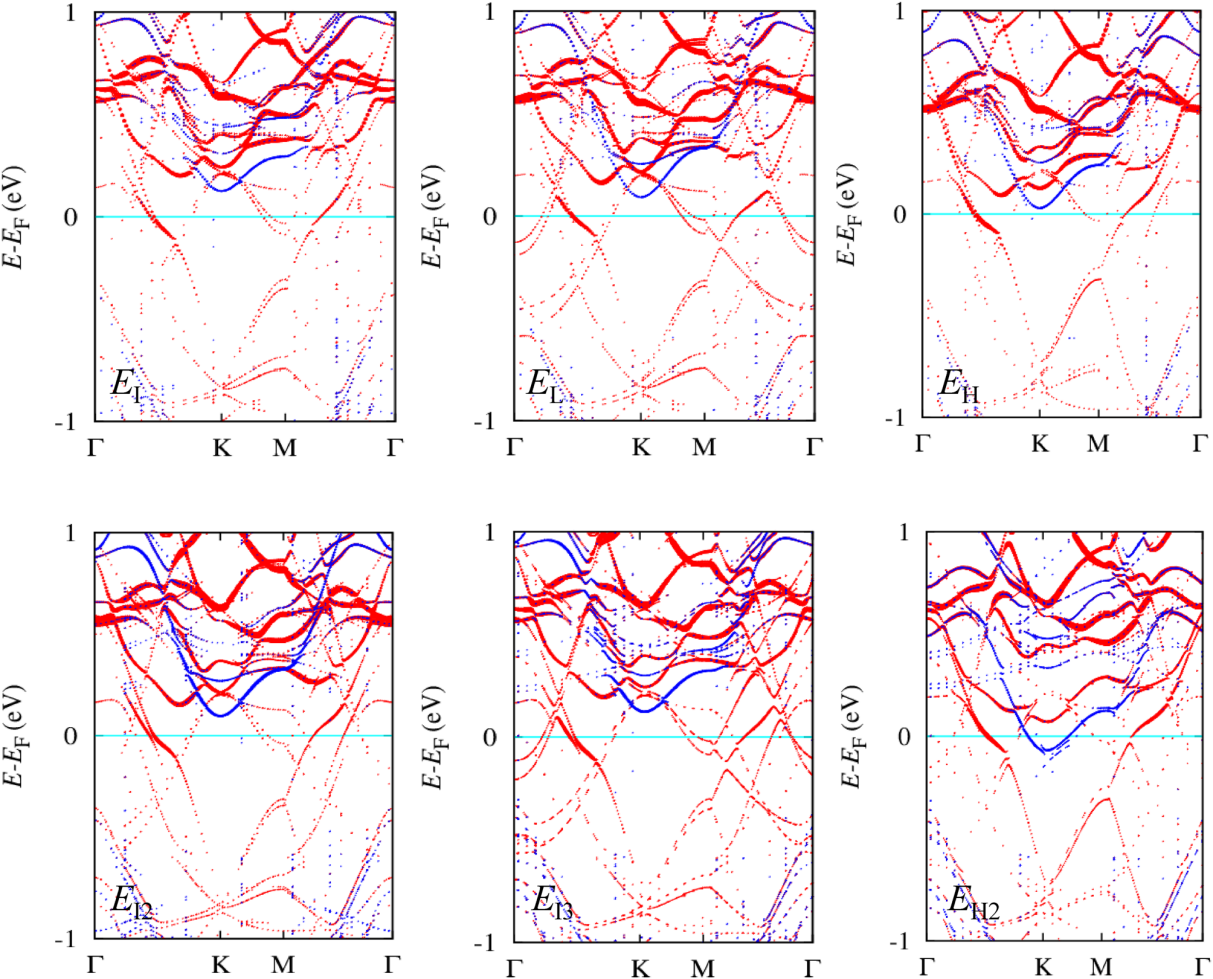
Band structures projected on a Cr atom for various stacked structures. The band structures are obtained by the noncollinear PBE + U + SOC method (*U* = 3.5 eV and *J* = 1.0 eV applied to Cr 3*d* orbitals). The red (blue) color is for spin-up (spin-down) bands; the color intensity corresponds to the projection magnitude.

**Figure 5:**
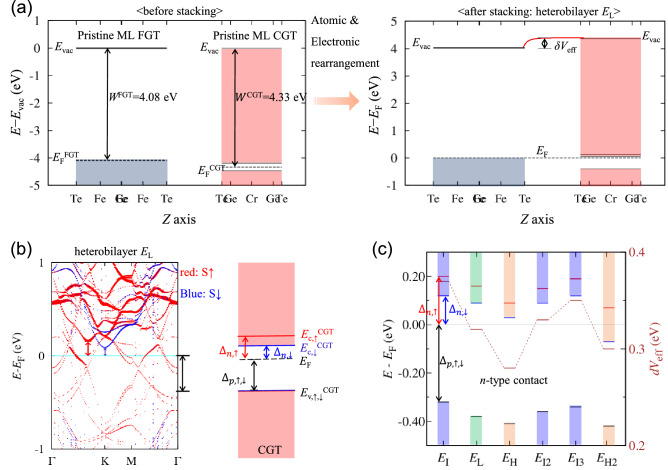
(**a**) Relative band alignment of the FGT and CGT layers before (left panel) and after stacking (right panel). Right after stacking, atomic and electronic rearrangements occur. *E*_vac_ and *E*_F_ denote the vacuum and Fermi levels, respectively. The effective potential difference, δ*V*_eff_, is noted at the interface. (**b**) Band structures for *E*_L_ (left panel) and schematic band edges of the CGT layer in the heterobilayer (right panel). The spin-up (red) and spin-down (blue) bands are projected onto a Cr atom; the color intensity corresponds to the projection magnitude. The *n*-type (${\Delta }_{n}$), and *p*-type (${\Delta }_{p}$) band edges are indicated by colored arrows. (**c**) Band edges of the CGT layer and effective potential difference, δ*V*_eff_, depending on the stacked structures.

**Figure 6:**
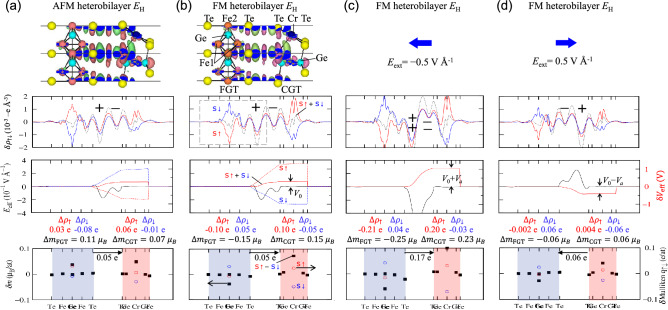
In-plane averaged spin-dependent electron density difference ($\updelta {\uprho }_{\uparrow ,\downarrow }\left(z\right)={\rho }_{\mathrm{HB}\uparrow ,\downarrow }\left(z\right)-{\rho }_{\mathrm{FGT}\uparrow ,\downarrow }(z)-{\rho }_{\mathrm{CGT}\uparrow ,\downarrow }(z)$, top panels), effective electric field, *E*_eff_, effective potential difference, δ*V*_eff_ (middle panels), magnetic moment variation ($\updelta m\left(\mathrm{z}\right)={m}_{\mathrm{HB}}\left(z\right)-{m}_{\mathrm{FGT}}\left(z\right)-{m}_{\mathrm{CGT}}(z)$), and spin-dependent Mulliken electron difference (bottom panels) of (**a**) the AFM FGT/CGT heterobilayer, (**b**) FM heterobilayer, (**c**) FM heterobilayer in a − 0.5 VÅ^−1^ negative electric field, and (**d**) FM heterobilayer in a 0.5 VÅ^−1^ positive electric field. The *x*-tics represent the *z*-components of atomic positions. The effective electron density difference (*δρ*_eff_(*z*) = *δρ*_↑_(*z*) + *δρ*_↓_(*z*)) is indicated by black dotted lines in the top panel. On the atomic structures in (**a**) and (**b**), the effective electron density difference with electron accumulation (magenta) and depletion (green) is depicted. The + and − symbols represent the electron depletion and accumulation at the interface in the top panel, respectively. The general signs of ${E}_{\mathrm{eff}}$ and $\updelta {V}_{\mathrm{eff}}$ for the positive charge are opposite to them calculated for the electron in this study. $\Delta {\rho }_{\uparrow \downarrow }$ denotes the spin-dependent total electron density difference per single layer in the heterobilayer, and $\Delta m$ represents the total magnetic moment variation per single layer. The red and blue open circles at Fe2 and Cr atoms indicate the spin-up and spin-down Mulliken electron differences, respectively.

Figure 4 shows the spin-up (red) and spin-down (blue) band structures projected on a Cr atom of all the stacked structures with the FM spin alignment. All the band structures exhibit spin-up hybridized bands crossing the Fermi level. However, depending on stacking, the position of the spin-down CBM of Cr bands close to the Fermi level varies. The origin of the band alignment based on stacked structures is shown in Fig. 5. The relative band alignment between the FGT and CGT layers before and after stacking is shown in Fig. 5a. Before stacking, the calculated work function of pristine ML CGT, 4.33 eV, is slightly larger than that of pristine ML FGT, 4.08 eV. After stacking, atomic and electronic rearrangements occur, especially near the interface. The electron transfer from the FGT to CGT layers causes an *n*-type CGT layer to be formed. When the work function of the metal is less than that of the *n*-type semiconductor, band bending at the interface generates an Ohmic contact between the metal and the *n*-type semiconductor. Our vdW heterobilayer, on the other hand, has the vdW interlayer space without any extra bonds between the two constituent layers, resulting in an abrupt interface. There is only the interface dipole at the interface, which is dictated by electron distributions. The interface dipole is defined by the associated effective potential difference at the interface, $\updelta {V}_{\mathrm{eff}}$. The electrostatic potential difference between the vacuum levels of the FGT and CGT layers in the heterobilayer can be used to determine $\updelta {V}_{\mathrm{eff}}$, which is reported in Table 2. In addition, $\updelta {V}_{\mathrm{eff}}$ is compared with that calculated using the electron density difference, and the relationship between $\updelta {V}_{\mathrm{eff}}$ and electron transfer is explored in Fig. 6.

**Table 2:** Spin-dependent band edges ${\Delta }_{n,\uparrow ,\downarrow }$ and ${\Delta }_{p,\uparrow ,\downarrow }$, the effective bandgap of the CGT layer, *E*_g_^CGT^, and effective potential difference, *δV*_eff_, obtained by the electrostatic potential difference between the vacuum levels of the FGT and CGT layers in the FM heterobilayer.

	*d*_inter_ (Å)	${\Delta }_{n,\uparrow }$(eV)	${\Delta }_{n,\downarrow }$(eV)	${\Delta }_{p,\uparrow ,\downarrow }$(eV)	*E*_g_^CGT^ (eV)	*δV*_eff_ (eV)
*E*_I_	3.39	0.20	0.12	0.32	0.44	0.35
*E*_L_	3.22	0.16	0.09	0.38	0.47	0.31
*E*_H_	3.78	0.09	0.03	0.41	0.44	0.28
*E*_I2_	3.33	0.15	0.09	0.36	0.45	0.33
*E*_I3_	3.31	0.19	0.12	0.34	0.46	0.35
*E*_H2_	3.71	0.07	− 0.07	0.42	0.39	0.30

The band edges of the *n*-type CGT layer in the heterobilayer were computed to quantify the band alignment. The spin-up (red) and -down (blue) bands of *E*_L_ projected on a Cr atom are shown in the left panel of Fig. 5b, with color intensity according to the projection magnitude. Colored arrows indicate the CBM (${E}_{\mathrm{C},\uparrow ,\downarrow }^{\mathrm{CGT}}$) and VBM (${E}_{\mathrm{V},\uparrow ,\downarrow }^{\mathrm{CGT}}$) of the original Cr bands relative to the Fermi level. The CGT layer’s schematic band edges are shown in the right panel of Fig. 5b. In metal/*n*-type semiconductor heterostructures, the *n*-type spin-up and spin-down band edges are specified as ${\Delta }_{n,\uparrow ,\downarrow }= {E}_{\mathrm{C},\uparrow ,\downarrow }^{\mathrm{CGT}}-{E}_{\mathrm{F}}$. In metal/*p*-type semiconductor heterostructures, the *p*-type spin-up and spin-down band edges are expressed as ${\Delta }_{p,\uparrow ,\downarrow }={E}_{\mathrm{F}}-{E}_{\mathrm{V},\uparrow ,\downarrow }^{\mathrm{CGT}}$. Because ${E}_{\mathrm{V},\uparrow }^{\mathrm{CGT}}={E}_{\mathrm{V},\downarrow }^{\mathrm{CGT}}$ in our case, ${\Delta }_{p,\uparrow }={\Delta }_{p,\downarrow }$. The effective bandgap in the CGT layer can be described as ${E}_{\mathrm{g}}^{\mathrm{CGT}}={\Delta }_{n,\downarrow }+{\Delta }_{p,\uparrow ,\downarrow }$ even if it is not a genuine bandgap owing to the metal wave function tail of the FGT layer up to the Fermi level. The band edges of the CGT layer and $\updelta {V}_{\mathrm{eff}}$ with respect to the various stacked structures are shown in Fig. 5c, and the estimated values for the band edges and ${E}_{\mathrm{g}}^{\mathrm{CGT}}$ are summarized in Table 2. According to Fig. 5c, the variations in band edges and $\updelta {V}_{\mathrm{eff}}$ across different stacked structures are similar, suggesting a significant correlation. For example, the magnitude order in ${\Delta }_{n,\uparrow ,\downarrow }$ is *E*_I_ > *E*_L_ > *E*_H_, which is the same as in $\updelta {V}_{\mathrm{eff}}$. In all the stacked structures, ${\Delta }_{p,\uparrow ,\downarrow }$ is larger than ${\Delta }_{n,\downarrow }$, confirming the *n*-type CGT, and the biggest variation in band edges across different structures is roughly 0.1 eV.

To study the spin/electron transfer and $\updelta {V}_{\mathrm{eff}}$ derived from the electron density difference, the spin-resolved valence electron density difference $\updelta {\uprho }_{\uparrow ,\downarrow }\left(z\right)={\rho }_{\mathrm{HB}\uparrow ,\downarrow }\left(z\right)-{\rho }_{\mathrm{FGT}\uparrow ,\downarrow }(z)-{\rho }_{\mathrm{CGT}\uparrow ,\downarrow }(z)$ was computed. Here, ${\rho }_{\mathrm{HB}\uparrow ,\downarrow }(z)$,${\rho }_{\mathrm{FGT}\uparrow ,\downarrow }(z)$, and ${\rho }_{\mathrm{CGT}\uparrow ,\downarrow }(z)$ are the spin-dependent in-plane-averaged electron densities of the heterobilayer, FGT-only, and CGT-only systems. In the case of FM heterobilayer, $\updelta {\uprho }_{\uparrow ,\downarrow }$ (top panel in Fig. 6b) illustrates that the spin-up density transfers from the FGT to CGT layers, and the spin-down density transfers from the CGT to FGT layers, resulting in the effective electron density difference (black dotted lines,${\delta \rho }_{\mathrm{eff}}\left(z\right)={\delta \rho }_{\uparrow }\left(z\right)+{\delta \rho }_{\downarrow }(z)$). A considerable electron transfer from Fe2 atoms to Cr atoms is observed in the atomic-structure figure via the bonding between Fe2 atoms and interface Te_FGT_ atoms, via the vdW spacing between interface Te_FGT_ atoms and interface Te_CGT_ atoms, and via the bonding between interface Te_CGT_ atoms and Cr atoms. Whereas the electron transfer from Fe1 atoms to Cr atoms is absent due to no direct bonding between Fe1 atoms and interface Te_FGT_ atoms. This electron-transfer pathway explains the hybridized band between Cr atoms and Fe2 atoms. In the interface region, the interface dipole appears as electron depletion and accumulation, represented by + and − signs, respectively, resulting in an effective electric field and a potential difference at the interface.

Specifically, the one-dimensional Gauss law can be used to compute the effective electric field, ${E}_{\mathrm{eff}}\left(z\right)$, and effective potential difference, $\updelta {V}_{\mathrm{eff}}(z)$, at the interface, as illustrated in the middle panel of Fig. 6b. ${Q}_{in}={\int }_{z0}^{z}\delta {\rho }_{\mathrm{eff}}\left(z\right)dz$ gives the electron density inside the gray dashed box in the top panel, where z_0_ is an arbitrary point *z* at the left side vacuum outside the FGT layer. Because the electric field at the left side vacuum is zero, ${E}_{\mathrm{eff}}\left(z\right)={Q}_{\mathrm{in}}(z){({\varepsilon }_{r}{\varepsilon }_{o})}^{-1}$ gives the electric field at location *z*, where ${\varepsilon }_{o}$ is the vacuum permittivity, and ${\varepsilon }_{r}$ is the position-dependent relative permittivity. Since ${\varepsilon }_{r}$ is around 1.0, 13.50, and $\infty$ in the vacuum, CGT, and FGT layers, respectively, the effective electric field exists only at the interface between the two layers. Finally, $\updelta {V}_{\mathrm{eff}}(z)$ for electrons at the interface may be calculated using $\updelta {V}_{\mathrm{eff}}\left(z\right)=-{\int }_{0}^{z}{E}_{\mathrm{eff}}\left(z\right)dz$; further, it is verified that $\updelta {V}_{\mathrm{eff}}(z)$ equals the sum of $\updelta {V}_{\mathrm{eff},\uparrow }(z)$ and $\updelta {V}_{\mathrm{eff},\downarrow }(z)$. The Gauss law yields $\updelta {V}_{\mathrm{eff}}$ values (*V*_o_ in Fig. 6b) of 0.28 eV, 0.32 eV, and 0.37 eV for *E*_H_, *E*_L_, and *E*_I_, respectively, which are extremely similar to the electrostatic potential differences of 0.28 eV, 0.31 eV, and 0.35 eV, respectively. Notably, the general signs for ${E}_{\mathrm{eff}}$ and $\updelta {V}_{\mathrm{eff}}$ defined for a positive charge are the inverse of those defined for the electron in this work.

The spin variation (${\Delta \rho }_{\uparrow ,\downarrow }={\rho }_{\mathrm{HB},\uparrow ,\downarrow }-{\rho }_{1\mathrm{L},\uparrow ,\downarrow })$ and the charge variation ($\Delta\uprho ={\Delta \rho }_{\uparrow }+{\Delta \rho }_{\downarrow })$ per single layer were quantitatively investigated using the spin-dependent Mulliken electron differences at the atomic sites, as shown in the bottom panel of Fig. 6b. The spin-up electron in the FGT layer moves to the spin-up electron in the CGT layer by 0.10 e. In contrast, the spin-down electron in the CGT layer moves to the spin-down electron in the FGT layer by 0.05 e, resulting in a 0.05 e effective spin-up electron transfer from FGT to CGT. Also, the magnetic moment variation per single layer, i.e., ${\Delta \rho }_{\uparrow }-{\Delta \rho }_{\downarrow }$ is $\Delta {m}_{\mathrm{FGT}}=-$ 0.15 ${\mu }_{\mathrm{B}}$ and $\Delta {m}_{\mathrm{CGT}}=$ 0.15 ${\mu }_{\mathrm{B}}$. In addition, the magnetic moment variation by the atomic site ($\updelta m$, solid black square) demonstrates that magnetic moments are slightly larger at Cr sites and slightly smaller at Fe2 sites than those of a single layer. The spin-down variation (blue open circle) contributes more to the magnetic moment changes at the Cr and Fe2 sites than the spin-up variation (red open circle).

On the other hand, the FGT layer’s $\updelta {\uprho }_{\uparrow ,\downarrow }$ in the AFM heterobilayer, as illustrated in the top panel of Fig. 6a, is reversed compared to that in the FM heterobilayer due to the FGT layer’s reversed spin-up and spin-down bands in the AFM heterobilayer. However, the electron distribution at the interface in the AFM heterobilayer is identical to that in the FM heterobilayer, resulting in similar ${E}_{\mathrm{eff}}$ and $\updelta {V}_{\mathrm{eff}}$. As demonstrated in the atomic-structure figure in Fig. 6a, the practical electron-transfer pathway in the AFM heterobilayer is comparable to that in the FM heterobilayer. According to the quantitative spin variation per single layer, complex spin transfer between FGT and CGT results in a 0.05 e effective electron transfer from FGT to CGT. Also, the magnetic moment variation per single layer is $\Delta {m}_{\mathrm{FGT}}=$ 0.11 ${\mu }_{\mathrm{B}}$ and $\Delta {m}_{\mathrm{CGT}}=$ 0.07 ${\mu }_{\mathrm{B}}$. The magnetic moments of both the Cr and Fe2 sites are slightly larger than those of a single layer.

### Electrostatic gating effect

**Figure 7:**
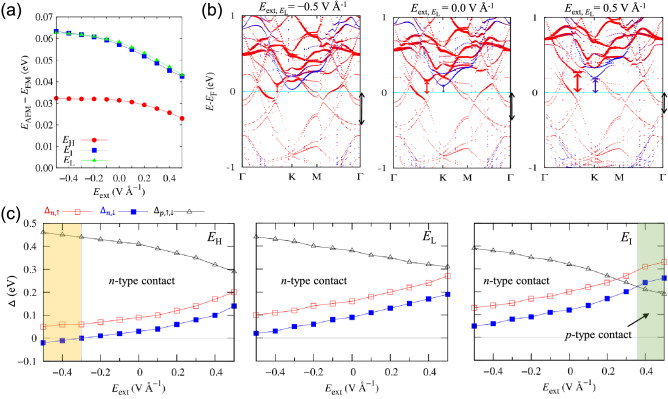
(**a**) Total energy difference between FM and AFM spin alignments between the FGT and CGT layers of *E*_H_, *E*_I_, and *E*_L_ heterobilayers with respect to the external electric field. (**b**) Band structures under the external electric field of − 0.5 VÅ^−1^ (left), 0 VÅ^−1^ (middle), and 0.5 VÅ^−1^ (right) for *E*_L_ structure. The spin-up (red) and spin-down (blue) bands are projected onto a Cr atom; the color intensity corresponds to the projection magnitude. The band-edge magnitudes relative to the Fermi level are indicated by colored arrows. (**c**) Spin-dependent *n*-type (${\Delta }_{n\uparrow \downarrow }$) and *p*-type (${\Delta }_{p\uparrow \downarrow }$) band-edge changes as a function of external electric field for *E*_H_ (left panel), *E*_L_ (middle panel), and *E*_I_ (right panel) heterobilayers.

This study considers positive and negative electric fields within 0.5 VÅ^−1^, perpendicular to the in-plane direction. When utilizing the PBE + U + SOC method with *U* = 3.5 eV and *J* = 1.0 eV to compare the total energies of the FM and AFM *E*_H_, *E*_I_, and *E*_L_ structures concerning the external electric field, FM structures are always more stable than AFM structures, as shown in Fig. 7a. Figure 6c,d show the spin/electron transfer for the FM *E*_H_ structure. We show only *E*_H_ results because the main effect of electrostatic gating on all stacked structures is almost comparable. Our results show that the external electric field mainly modulates the spin-majority spin transfer between FGT and CGT. As shown in Fig. 6c, the variation of $\updelta {\rho }_{\uparrow ,\downarrow }$ increases when a negative electric field of − 0.5 VÅ^−1^ is introduced. Quantitatively, the spin-up electron moves from FGT to CGT by 0.21 e, whereas the spin-down electron moves from CGT to FGT by 0.03 e, resulting in a 0.17 e effective spin-up electron transfer from FGT to CGT. As a result, the magnetic moment variation per single layer increases, with $\Delta {m}_{\mathrm{FGT}}= -$ 0.25 ${\mu }_{\mathrm{B}}$ and $\Delta {m}_{\mathrm{CGT}}=$ 0.23 ${\mu }_{\mathrm{B}}$, respectively. More enhanced $\updelta m$ at Cr sites and more reduced $\updelta m$ at Fe2 sites are also induced by the negative electric field. In the interface region, the negative electric field promotes electron depletion (++) and accumulation (−−), resulting in larger ${E}_{\mathrm{eff}}$ and $\updelta {V}_{\mathrm{eff}}$ (*V*_o_ + *V*_a_, where *V*_a_ = 0.76 eV in Fig. 6c).

In contrast, the variation of $\updelta {\rho }_{\uparrow ,\downarrow }$ decreases under a positive electric field of 0.5 VÅ^−1^, as shown in Fig. 6d Consequently, the spin-up electron transfer is relatively tiny, whereas the spin-down electron transfer from CGT to FGT is 0.06 e, resulting in a 0.06 e effective spin-down electron transfer from the CGT to FGT layers. This spin transfer results in less increased and decreased $\updelta m$ at Cr and Fe2 sites, respectively, and reduces the magnetic moment variation per single layer. The positive electric field, especially in the interface region, generates even the opposite electron distribution, revealing the opposite action of the negative electric field. In particular, ${E}_{\mathrm{eff}}$ and $\updelta {V}_{\mathrm{eff}}$ (*V*_o_ − *V*_a_, where *V*_a_ = 0.70 eV in Fig. 6d) at the interface have the opposite sign.

The FGT/CGT heterobilayer is analogous to a Schottky diode in device performance, except for the opposite electron distribution in the interface. In a Schottky diode depletion zone, electron accumulation occurs near the metal, and electron depletion occurs near the n-type semiconductor. However, the electron distribution in our heterobilayer is reversed. A negative electric field to the heterobilayer, i.e., reverse bias, is achieved by connecting the battery’s positive terminal to CGT and the negative terminal to FGT. The positive terminal to CGT allows FGT’s electrons to migrate more to CGT, resulting in a stronger interface dipole (middle panel in Fig. 6c) and CGT’s lower energy-band shift relative to the Fermi level. Furthermore, as illustrated in the left panel of Fig. 7b, the CGT’s lower energy-band shift defines narrower *n*-type band edges but wider *p*-type band edges.

In contrast, a positive electric field, i.e., forward bias, is achieved by connecting the battery positive terminal to FGT and the negative terminal to CGT. The positive terminal to FGT causes CGT’s electrons to move to FGT, resulting in a smaller or inverted interface dipole (middle panel in Fig. 6d). On the other hand, the negative terminal to CGT leads to the CGT’s higher energy-band shift relative to the Fermi level. Furthermore, as shown in the right panel of Fig. 7b, the CGT’s higher energy-band shift sets wider *n*-type band edges but narrower *p*-type band edges.

Figure 7c illustrates how the band edges change with the external electric field. As the external electric field increases, *n*-type band edges widen, and *p*-type band edges narrow, regardless of the stacked structures of *E*_H_, *E*_I_, and *E*_L_. However, slightly different band-edge values depending on the stacked structures cause differences in the contact type as the external electric field changes. In the *E*_L_ heterobilayer, the *n*-type contact is maintained in the range of − 0.5 VÅ^−1^ to 0.5 VÅ^−1^. In the *E*_H_ heterobilayer, the *n*-type contact changes to a particular contact (${\Delta }_{n,\downarrow }<0$) below − 0.3 VÅ^−1^. In the *E*_I_ heterobilayer, the *n*-type contact changes to *p*-type contact above 0.36 VÅ^−1^. These differences, depending on the different stacked structures, originate from the different initial potential differences ($\updelta {V}_{\mathrm{eff}}$ when there is no external electric field) due to different asymmetric interface structures (Supplementary Information).

## Conclusions

We presented the structural, electronic, and magnetic properties of layered FGT/CGT twisted moiré heterobilayers and the electrostatic gating effect. Our results show that the moiré heterobilayer with a twist angle of 30° is an FM metal with an *n*-type CGT layer regardless of various stacked structures. In the spin transfer of the FM heterobilayer, an effective spin-up electron transfer occurs from FGT to CGT. Furthermore, the spin-up hybridized bands between Cr and Fe2 atoms crossing the Fermi level are found only in the FM heterobilayer, not in the AFM heterobilayer, independent of stacking. It also turns out that the band alignment of the CGT layer is explained by the effective potential difference at the interface. Our findings further reveal that, in the FM heterobilayer, the external electric field regulates the spin-up electron transfer between the FGT and CGT layers and affects the band edges of the CGT layer. Our study could provide helpful information for understanding the effects of stacking, spin alignment, spin transfer, and electrostatic gating in the magnetic 2D vdW metal/semiconductor heterobilayer.

## Supplementary Information

Supplementary Information.

## Data Availability

All data generated or analyzed during this study are included in this published article.
