# Supplementary material for: Hybridized bands and stacking-dependent band edges in ferromagnetic Fe3GeTe2/CrGeTe3 moiré heterobilayer
Source: Sci Rep. 2022 Mar 24;12:5101. doi: 10.1038/s41598-022-08785-x (PMC8948266; doi:10.1038/s41598-022-08785-x)
Supplement: Supplementary file 1 — Supplementary Information. [file 41598_2022_8785_MOESM1_ESM.pdf]

Supplementary Information

Title: Hybridized bands and stacking-dependent band edges in ferromagnetic Fe_3GeTe_2/$CrGeTe_3$ moiré heterobilayer

Eunjung Ko*

Korea Institute for Advanced Study, Seoul 02455, Korea

Contents

1.1 Construction of twisted model heterostructures

1.2 *U*-dependent band edges of the $CrGeTe_3$ layer in ferromagnetic $Fe_3GeTe_2/CrGeTe_3$ heterobilayers in the absence or presence of the external electric fields

1.1 Construction of twisted model heterostructures

On pages 6 and 7 of our manuscript, we explained how the commensurate moiré heterobilayer structures for computations were generally generated. Moire supercells within a small biaxial strain Δ can be obtained by solving the Diophantine equation of $|\vec{L}_{\mathrm{CGT},1}|_{\pm\Delta} = |\vec{L}_{\mathrm{FGT},1}|_{\mp\Delta}$. Δ is an unavoidable parameter due to the lattice mismatch between FGT and CGT. A moiré supercell for CGT and FGT is an in-plane rotated supercell with a different rotation angle, respectively. Thus, the twist angle is determined by the difference of in-plane rotation angles, i.e., twist angle ϕ = 16.1° – 4.7° = 11.4° in Fig. S1(a) and ϕ = 30.0° – 0.0° = 30.0° in Fig. S1(b).

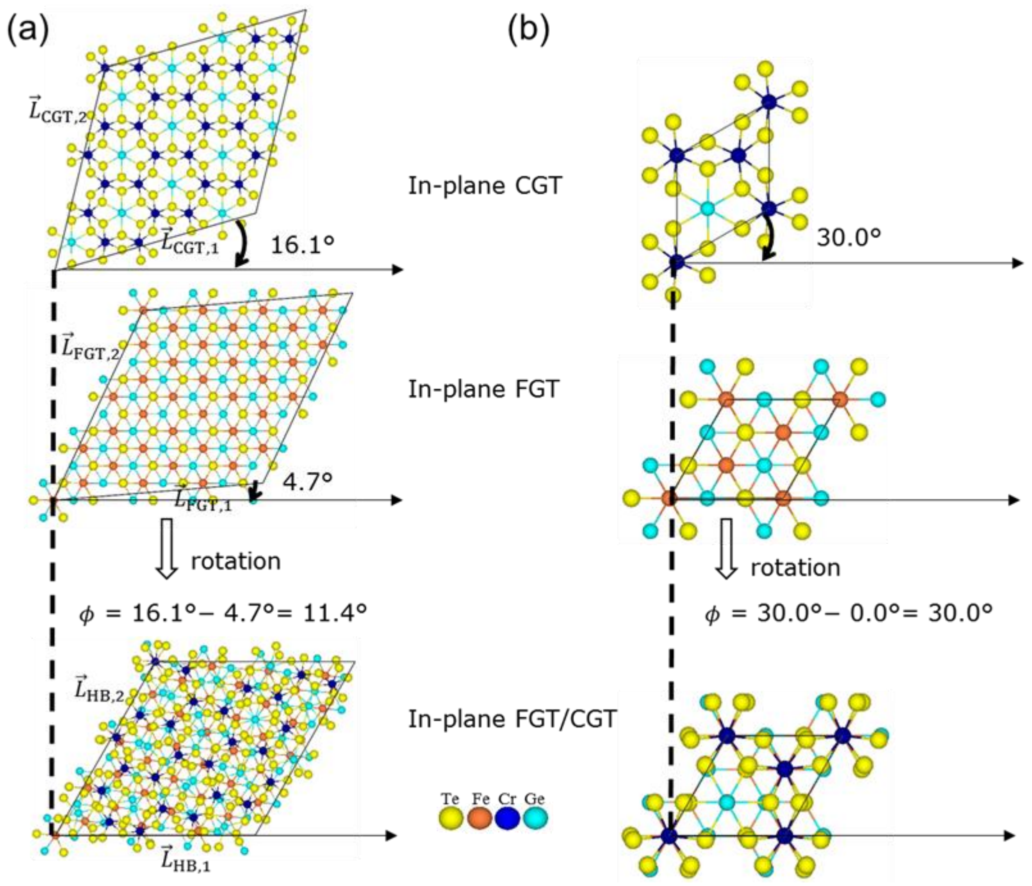

Fig. S1. Twisted moiré-heterobilayer construction of our model structures with twist angles of (a) 11.4° and (b) 30.0°.

1.2 U-dependent band edges of the $CrGeTe_3$ layer in ferromagnetic $Fe_3GeTe_2/CrGeTe_3$ heterobilayers in the absence or presence of the external electric fields

In our manuscript, we have shown the calculation results using U = 3.5 eV and J = 1 eV. However, we have confirmed that the calculation results using U = 3 or 4 eV and J = 1 eV showed similar trends to those using U = 3.5 eV and J = 1 eV. Fig. S2(a) shows the U-dependent n-type ($\Delta_{n\uparrow\downarrow}$) and p-type ($\Delta_{p\uparrow\downarrow}$) band edges of the CGT layer for the E_I, E_L, and E_H heterobilayers when there is no external electric field (E_{ext}). The order of band-edge alignment between E_I, E_L, and E_H is similar regardless of U values. The U-dependent difference is that $\Delta_{n\downarrow}$ and $\Delta_{p\uparrow\downarrow}$ shift higher in energy as U increases, while $\Delta_{n\uparrow}$ remains the same. Thus, the interval between $\Delta_{n\downarrow}$ and $\Delta_{n\uparrow}$ becomes smaller as U increases. Figs. S2(b)−(d) show the U-dependent band-edge changes as a function of E_{ext} when (b) U = 3 eV, (c) 3.5 eV, and (d) 4 eV and J = 1 eV. The overall trends with respect to E_{ext} are similar regardless of U values. The U-dependent interval difference between $\Delta_{n\downarrow}$ and $\Delta_{n\uparrow}$ is also observed in the presence of E_{ext}. It is also found that the E_{ext} range, where $\Delta_{n\downarrow}$ is negative (orange-colored region), decreases within the range of ± 0.5 V Å^{-1} as U increases, while the E_{ext} range of the p-type-contact region (green-colored region) increases.

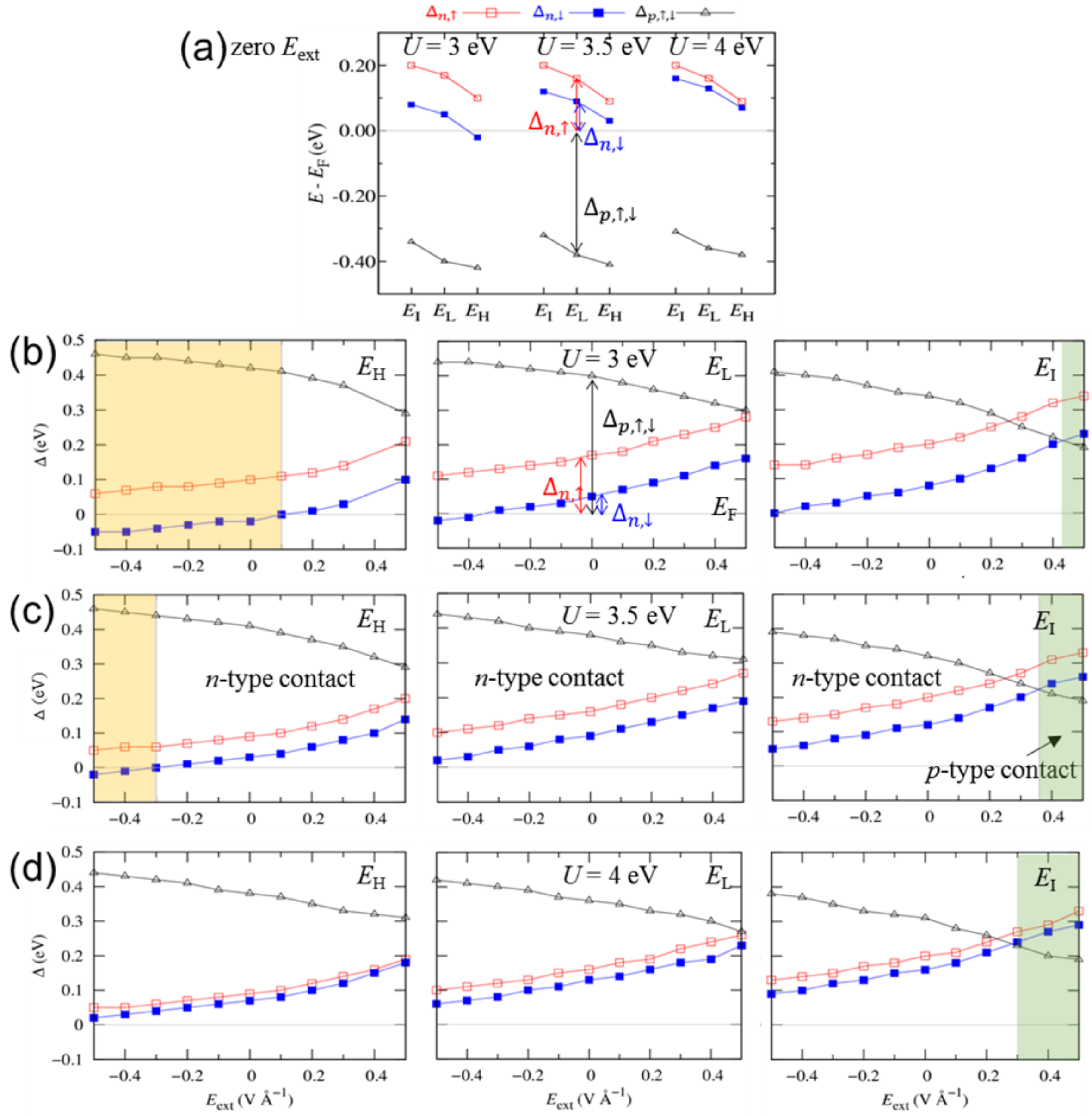

Fig. S2. (a) U-dependent n-type ($\Delta_{n\uparrow\downarrow}$) and p-type ($\Delta_{p\uparrow\downarrow}$) band edges of the CGT layer for E_I, E_L, and E_H heterobilayers when there is no external electric field (E_{ext}). U-dependent band-edge changes as a function of E_{ext} for E_H (left panel), E_L (middle panel), and E_I (right panel) heterobilayers when (b) U = 3 eV, (c) 3.5 eV, and (d) 4 eV. In (a)−(d), the J value is 1 eV. In (b)−(d), the orange-colored region indicates the E_{ext} range in which the $\Delta_{n\downarrow}$ is negative, and the green-colored region represents the p-type-contact region.
